# The association between time-mean serum uric acid levels and the incidence of chronic kidney disease in the general population: a retrospective study

**DOI:** 10.1186/s12882-018-0982-6

**Published:** 2018-07-31

**Authors:** Meiyu Ye, Kang Hu, Juan Jin, Diandian Wu, Peiying Hu, Qiang He

**Affiliations:** 1 Department of Nephrology, Zhejiang Provincial People’s Hospital, People’s Hospital of Hangzhou Medical College, No. 158 Shangtang Road, Hangzhou, 310014 Zhejiang Province People’s Republic of China; 2 The Second Clinical Medical College, Zhejiang Chinese Medical University, No. 548 Binwen Road, Hangzhou, 310053 Zhejiang Province People’s Republic of China; 3 Health Promotion Center, Zhejiang Provincial People’s Hospital, No. 158 Shangtang Road, Hangzhou, 310014 Zhejiang Province People’s Republic of China

**Keywords:** Time-mean, Serum uric acid, Chronic kidney disease, Renal function, Retrospective study

## Abstract

**Background:**

Investigations on the role of the time-mean serum uric acid (SUA) value in determining the risk of chronic kidney disease (CKD) are limited. We investigated whether the time-mean SUA value indicates the risk of CKD, and explored associations of the baseline and time-mean SUA levels with kidney function decline and incident CKD in a healthy population.

**Methods:**

We initiated an inhabitant-based cohort study between January 2011 and December 2016. All participants completed a yearly medical check-up at the Zhejiang Province People’s Hospital and had baseline estimated glomerular filtration rates (eGFR) > 60 ml/min/1.73m^2^. The SUA level and eGFR were assessed every year in the follow-up period. A multivariate adjusted binary logistic regression analysis and Cox proportional hazards models were used to evaluate the risk of newly-developed CKD among different stratified groups.

**Results:**

During the 6-year follow-up period, 227 (4.4%) participants developed CKD. In multivariable-adjusted analyses, the odds ratio (OR) for new-onset CKD increased, with higher time-mean SUA levels than at baseline (OR: 1.00 [reference], 2.709 [95% confidence interval: 1.836–5.293], 3.754 [1.898–7.428], and 7.462 [3.694–15.073]). After adjustment for potential cofounders, a multivariate Cox proportional hazard model showed that a higher SUA increased the risk of developing CKD (the adjusted hazard ratios of the highest and lowest quartiles for the baseline and time-mean SUA levels were 1.689 [1.058–2.696] and 6.320 [3.285–12.159], respectively).

**Conclusion:**

An increased time-mean and single SUA value were independently associated with an increased likelihood of eGFR decline and development of new-onset CKD in the general population.

## Background

Recently, with the gradual change of modern lifestyles and diets, the incidence of hyperuricemia is increasing. Uric acid (UA) is preponderantly excreted by the kidney; hence, aberrant production or clearance by the kidney, such as overproduction or underexcretion, can increase the UA levels [1]. A dramatic rise in the serum UA levels (SUA) was originally proposed as the cause of gout [2]. Based on observations of gout patients, Mahomed et al. hypothesized that hyperuricemia is a possible mediator of hypertension [3]. Subsequently, Haig suggested that UA could lead to many diseases in addition to gout, such as rheumatism, hypertension, diabetes, and chronic kidney disease (CKD) [4]. Since then, numerous epidemiologic studies have successively indicated that there is a relationship between elevated UA levels and metabolic syndrome [5], renal disease [5, 6], hypertension [7], and cardiovascular disease (CVD) [6, 8].

CKD has emerged as a primary public health problem worldwide [9], and the epidemic prevalence has doubled in the past several decades [10], particularly in China [11] and other developing countries [10]. CKD can lead to not only end-stage renal disease (ESRD), but also complications related to renal impairment and an increased risk of CVD [9].

As the diagnostic techniques and aggressive treatment strategies have developed, renewed attention has been given to elevated SUA levels. The relationship between the UA level and development of CKD has been supported by expanding epidemiologic and experimental evidence. Researchers have investigated the role of UA not only as a potential marker of renal dysfunction [6, 12–14], but also as a significant pathogenic factor that is involved in the development of renal disease [12, 15–18]. Basic experimental studies have confirmed that the potential mechanisms of renal injury due to hyperuricemia induce inflammation, afferent arteriopathy [16], and endothelial dysfunction [18]; activate the renin-angiotensin-aldosterone system (RAAS) [17] and cyclooxygenase-2 (COX-2) expression; and impair oxidative metabolism [12], among others. In contrast, many large epidemiologic studies have demonstrated that elevated SUA levels were an independent risk factor for the worsening of renal function and incident CKD in the general population [19, 20], after adjusting for age, sex, race, diabetes, systolic blood pressure (SBP), hypertension, lipids, history of underlying disease, baseline estimated glomerular filtration rate (eGFR), living habits, and other factors [19–22]. Furthermore, a prospective cohort study of healthy people suggested that there was a U-shaped association between UA and the loss of kidney function. This means that both low and high UA levels may predict a decline in kidney function [23]. However, many cohort studies have shown conflicting results, indicating that there was no significant association between an increased SUA level and CKD progression [24–26], especially after adjustment for confounders.

Therefore, to avoid the instability of a single test, which might not be sufficient for identifying patients at risk, we aimed to investigate whether the time-mean SUA value indicates the risk of CKD, and explored the association of the baseline and time-mean SUA levels with kidney function decline and incident CKD in an ostensibly healthy population.

## Methods

The study design is a general population-based retrospective observational study of Chinese(ranging in age from 25 to 85 years). Participants work in the urban district of Hangzhou and participate an annual medical checkup at the Zhejiang Province People’s Hospital between 2011 and 2016.

### Study population

**Fig. 1:**
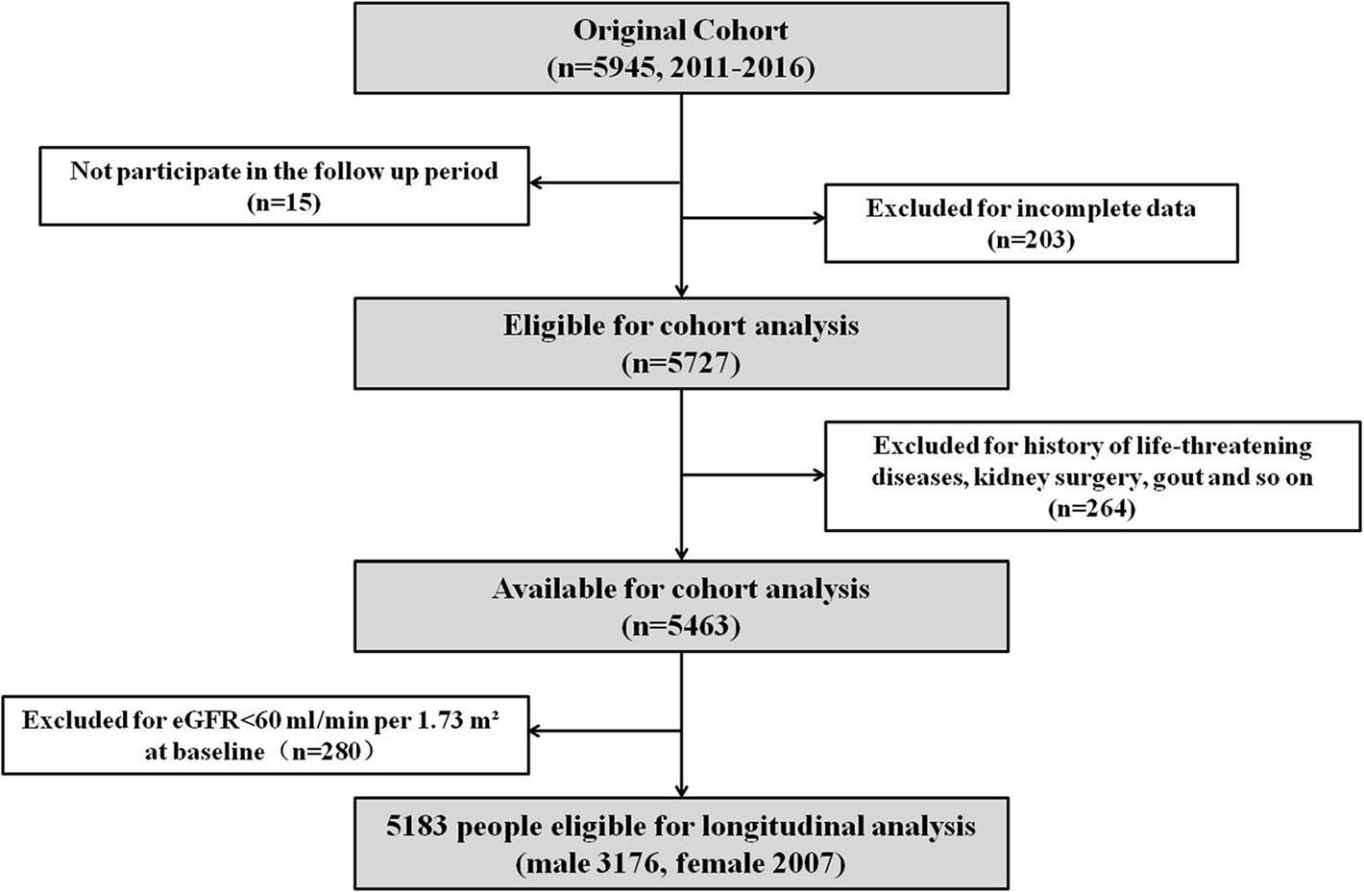
Flow diagram of study population. eGFR, estimated glomerular filtration rate

As described in Fig. 1, initially, a total of 5945 participants had available data at baseline were included. Among them, 15 individuals were not able to participate in the follow up period. We further excluded 203 participants for incomplete data. This left 5727 subjects eligible for analysis. Furthermore, 264 Individuals were excluded for the following past history: cardiovascular diseases(angina or acute myocardial infarction), cerebrovascular disease(cerebral apoplexy, cerebral infraction or cerebral hemorrhage), liver function damage, infectious disease, malignancies, kidney surgery, gout or other life-threatening diseases. For the remaining 5463 subjects, 280 individuals were excluded due to baseline eGFR < 60 ml/min/1.73 m^2^. Finally, the total number of eligible was 5183 participants.

### Measurements and data collection

The baseline characteristics of the participants included age, gender, physical examination, biochemical measurements and medical history evaluation. Weight and height were measured while participants were wearing light clothing and without shoes. Body mass index (BMI) was calculated as weight (kg) divided by the square of the height (m). Systolic and diastolic blood pressures (SBP/DBP) were measured by an automated sphygmomanometer while the subjects were in a seated position after resting for 5 min. Blood specimens were drawn after an overnight fast of more than 12 h. Biomedical parameters including blood urea nitrogen (BUN), fasting plasma glucose (FPG), serum total cholesterol, triglyceride, high-density lipoprotein cholesterol levels (HDLC), low-density lipoprotein cholesterol levels (LDLC), were measured by biochemical automated enzymatic analyzer in the clinical laboratory at Zhejiang Province People’s Hospital. All the covariates were measured once at baseline. SUA and serum creatinine (Scr) were measured at baseline and subsequent every year during the follow-up period by the uricase method and enzymatic method. The time-mean SUA value for each participant was calculated for reflecting the trend of data concentration, which could be preferable to estimate the accuracy of SUA levels in spite of varying instability factor. Renal function was calculated by using the formula of estimated glomerular filtration Rate (eGFR) (ml/min/1.73m^2^) =186 × SCr^-1.154^ × age^-0.203^ (× 0.742, if female), which was derived from the simplified Modification of Diet in Renal Disease (MDRD) study for Chinese people [27].

### Definition and primary outcome

Overweight was defined as a BMI between 24 and 28 kg/m^2^ and obesity was defined as a BMI ≥ 28 kg/m^2^ at the initial examination. Hypertension was defined as SBP ≥ 140 mmHg or DBP ≥ 90 mmHg or by self-reported history of hypertension. Diabetes was defined as a FPG level ≥ 7.0 mmol/L (126 mg/dl) or non-fasting glucose≥11.1 mmol/L (200 mg/dl) or by self-reported history of diabetes. Hyperuricemia was defined as SUA level > 7 mg/dl (420 μmol/L) in males and > 6 mg/dl (360 μmol/L) in females. According to the assessment and regulation of Chronic Kidney Disease, which derived from the Kidney Disease: Improving Global Outcomes (KDIGO) 2012 Clinical Practice Guideline [28], the new incidence of CKD event was defined as eGFR< 60 ml/min/1.73m^2^ in the follow up period. For individuals who had more than one CKD event during the follow-up period, only the first event was included in our statistical analysis.

### Statistical analysis

Statistical results were carried out by using IBM SPSS, version 20 and Stata, version 14. All values of results were two-tailed, and *P*-values < 0.05 were considered to be statistically significant. Baseline characteristics with normal distribution were reported as the mean (±standard deviation, SD) and percentage. Non-normally distributed variables were presented as the median with interquartile range (IQR). Independent-samples T-test was used to compare those with and without new incidence of CKD. The statistical differences between the baseline characteristics in relation to the quartiles of SUA levels was analyzed by using One-way ANOVA test for continuous variables, and Chi-square test for discrete variables. Multivariate linear regression analysis was used to evaluate the association between SUA levels and eGFR change. Multivariable-adjusted logistic regression analyses were employed to estimate odds ratio (OR) and 95% confidence interval (95% CI) for the new-onset CKD. Cox proportional hazard models were used to examine hazard ratio (HR) and 95% CI for the risk of having CKD after adjusting for potential confounding factors.

## Results

A total of 5183 subjects met the inclusion criteria and were selected for the present analysis. The flow diagram of study population is described in Fig. 1.

### Baseline characteristics

**Fig. 2:**
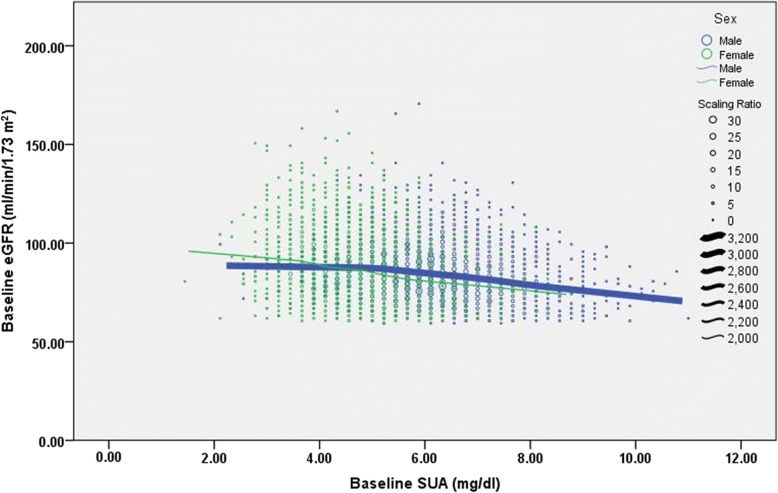
The relationship between serum uric acid levels and eGFR at first visit by gender

The distribution of the SUA levels at baseline based on sex is shown in Fig. 2. The baseline characteristics of the UA groups that were sex-stratified into quartiles are shown in Table 1 and Table 2. Overall, the majority of the participants were middle-aged. The median age of the patients in the study was 48 years (interquartile range [IQR]: 24) years for men and 49 (IQR: 22) years for women. The average SUA was 6.3 mg/dl for men and 4.7 mg/dl for women, and was greater in men than in women for all quartiled UA groups. The average eGFR was 84.9 ml/min/1.73m^2^ for men and 88.3 ml/min/1.73m^2^ for women. The correlation between these various variables and the UA level at baseline was similar in both men and women. Participants in quartile 4 (UA level > 6.9 mg/dl for men, and > 5.2 mg/dl for women) had a higher BMI and BP, total cholesterol, triglyceride, HDLC, LDLC, BUN, and FPG levels than those in quartile 1 (UA ≤ 5.5 mg/dl for men, and ≤ 4.0 mg/dl for women). This group also had a higher prevalence of patients who were overweight, obese, or hypertensive, as well as a lower eGFR, than those in quartile 1. However, subjects with higher SUA levels were more likely to be male than female, and the prevalence of overweight, obese, hypertensive, and diabetic patients, as well as the prevalence of increased BMI and BP, triglyceride, and FPG levels, was significantly higher in men than in women.

**Table 1:** Baseline characteristics of subjects by uric acid quartiles (Q1-Q4) at baseline (male)

Serum uric acid(mg/dl)	Male	Q1 (≤5.5)	Q2 (> 5.5, ≤6.2)	Q3 (> 6.2, ≤6.9)	Q4 (> 6.9)	*P* value*
N	3176	817	794	718	847	
Age (years)	48 (24)	48 (26)	44 (22)	47 (23)	50 (23)	0.001
BMI (kg/m^2^)	24.0±2.9	22.8±2.8	23.7±2.7	24.3±2.8	25.0±2.9	< 0.001
Overweight (24 ≤ BMI < 28) (%)	1329 (41.8)	233 (28.5)	311 (39.2)	332 (46.2)	453 (53.5)	< 0.001
Obesity (BMI ≥ 28) (%)	249 (7.8)	33 (4.0)	41 (5.2)	67 (9.3)	108 (12.8)	< 0.001
Systolic BP (mmHg)	128.6±17.4	126.9±17.7	127.2±17.2	129.3±17.5	130.9±16.8	< 0.001
Diastolic BP (mmHg)	78.0±10.8	76.2±10.8	77.2±11.1	78.6±10.4	79.9±10.7	< 0.001
Total cholesterol (mmol/L)	4.8±0.9	4.7±0.9	4.7±0.9	4.8±0.9	5.0±0.9	< 0.001
Triglycerides (mmol/L)	1.6±1.3	1.3±1.2	1.4±1.0	1.6±1.0	2.0±1.7	< 0.001
HDL cholesterol (mmol/L)	1.2±0.3	1.3±0.3	1.2±0.2	1.2±0.2	1.1±0.2	< 0.001
LDL cholesterol (mmol/L)	2.8±0.7	2.7±0.7	2.8±0.7	2.9±0.8	2.9±0.8	< 0.001
BUN (mmol/L)	5.2±1.1	5.1±1.2	5.2±1.1	5.3±1.1	5.3±1.1	0.031
Fasting plasma glucose (mmol/L)	5.3±0.9	5.3±1.1	5.2±0.8	5.3±0.9	5.3±0.8	0.019
eGFR (ml/min/1.73m^2^)	84.9±13.7	88.2±13.7	86.2±13.7	84.9±13.9	80.6±12.3	< 0.001
Serum uric acid (mg/dl)	6.3±1.2	4.9±0.5	5.9±0.2	6.5±0.2	7.7±0.7	< 0.001
Hypertension (%)	560 (17.6)	125 (15.3)	114 (14.4)	137 (19.1)	184 (21.7)	< 0.001
Diabetes (%)	136 (4.3)	43 (5.3)	30 (3.8)	27 (3.8)	36 (4.3)	0.409
New incident CKD (%)	139 (4.4)	22 (2.7)	22 (2.8)	31 (4.3)	64 (7.6)	< 0.001

*P value by ANOVA for continuous variables and chi square test for categorical variables

*BMI* body mass index, *eGFR* estimated glomerular filtration rate, *BUN* blood urea nitrogen, *HDL* high-density lipoprotein, *LDL* low-density lipoprotein, *eGFR* estimated glomerular filtration rate, *CKD* chronic kidney disease. Data were expressed as mean ± SD, median (interquartile range), or percentage (%)

**Table 2:** Baseline characteristics of subjects by uric acid quartiles (Q1-Q4) at baseline (female)

Serum uric acid(mg/dl)	Female	Q1 (≤4.0)	Q2 (> 4.0, ≤4.5)	Q3 (> 4.5, ≤5.2)	Q4 (> 5.2)	*P* value*
N	2007	548	440	524	495	
Age (years)	49 (22)	45 (18)	46 (19)	48 (23)	58 (25)	< 0.001
BMI (kg/m^2^)	22.1±2.8	21.1±2.6	21.7±2.5	22.1±2.7	23.5±3.1	< 0.001
Overweight (24 ≤ BMI < 28) (%)	402 (20.0)	73 (13.3)	62 (14.1)	99 (18.9)	168 (33.9)	< 0.001
Obesity (BMI ≥ 28) (%)	62 (3.1)	5 (0.9)	6 (1.4)	16 (3.1)	35 (7.1)	< 0.001
Systolic BP (mmHg)	120.6±19.1	116.4±17.2	118.3±16.9	120.1±19.3	128.0±20.9	< 0.001
Diastolic BP (mmHg)	71.7±11.0	69.8±10.2	70.9±10.6	71.5±10.7	74.7±11.8	< 0.001
Total cholesterol (mmol/L)	4.9±0.9	4.7±0.9	4.8±0.9	4.9±0.9	5.2±1.0	< 0.001
Triglycerides (mmol/L)	1.2±0.8	1.0±0.6	1.0±0.5	1.2±0.9	1.5±1.0	< 0.001
HDL cholesterol (mmol/L)	1.5±0.3	1.5±0.3	1.5±0.3	1.4±0.3	1.4 ±0.3	< 0.001
LDL cholesterol (mmol/L)	2.8±0.8	2.6±0.7	2.7±0.7	2.8±0.8	3.0±0.8	< 0.001
BUN (mmol/L)	4.7±1.1	4.5±1.1	4.6±1.1	4.6±1.1	5.0±1.1	< 0.001
Fasting plasma glucose (mmol/L)	5.1±0.7	5.0±0.6	5.1±0.6	5.1±0.6	5.3±0.8	< 0.001
eGFR (ml/min/1.73m^2^)	88.3±17.4	92.7±17.8	89.4±17.7	88.3±17.1	82.4±15.2	< 0.001
Serum uric acid (mg/dl)	4.7±1.0	3.5±0.4	4.3±0.1	4.8±0.2	5.9±0.6	< 0.001
Hypertension (%)	288 (14.3)	39 (7.1)	49 (11.1)	75 (14.3)	126 (25.5)	< 0.001
Diabetes (%)	61 (3.0)	5 (0.9)	14 (3.2)	16 (3.1)	27 (5.5)	< 0.001
New incident CKD (%)	88 (4.4%)	9 (1.6%)	11 (2.5%)	21 (4.0%)	47 (9.5%)	< 0.001

*P value by ANOVA for continuous variables and chi square test for categorical variables

*BMI* body mass index, *eGFR* estimated glomerular filtration rate, *BUN* blood urea nitrogen, *HDL* high-density lipoprotein, *LDL* low-density lipoprotein, eGFR estimated glomerular filtration rate, *CKD* chronic kidney disease. Data were expressed as mean ± SD, median (interquartile range), or percentage (%)

A comparison of the baseline characteristics of participants with and without new-onset CKD is shown in Table 3. During 6 years of follow-up, 227 patients (4.4%) experienced CKD. Participants who developed CKD were much older and more likely to be obese and have hyperuricemia, hypertension, or diabetes than those without CKD. The baseline and time-mean serum UA levels were both significantly higher among participants with new-onset CKD than in those without (6.24 ± 1.41 mg/dl and 5.59 ± 1.33 mg/dl [*P* < 0.001], and 6.39 ± 1.29 mg/dl and 5.64 ± 1.28 mg/dl [*P* < 0.001], respectively).

**Table 3:** Baseline characteristics of participants relative to development of CKD during the 6-year follow-up period

	CKD	Without CKD	*P* value*
N	227	4956	
Male (%)	139 (61.2)	3037 (61.3)	0.989
Age (years)	72 (23)	47 (22)	< 0.001
BMI (kg/m^2^)	24.3±2.8	23.2±3.0	< 0.001
Overweight (24 ≤ BMI < 28) (%)	110 (48.5)	1621 (32.7)	< 0.001
Obesity (BMI ≥ 28) (%)	22 (9.7)	289 (5.8)	0.017
Systolic BP (mmHg)	139.8±20.8	124.9±18.1	< 0.001
Diastolic BP (mmHg)	80.6±12.0	75.3±11.2	< 0.001
Total cholesterol (mmol/L)	5.1±1.0	4.8±0.9	< 0.001
Triglycerides (mmol/L)	1.7±1.3	1.4±1.1	0.001
HDL cholesterol (mmol/L)	1.3±0.3	1.3±0.3	0.048
LDL cholesterol (mmol/L)	3.0±0.8	2.8±0.8	< 0.001
BUN (mmol/L)	5.7±1.2	5.0±1.1	< 0.001
Fasting plasma glucose (mmol/L)	5.5±1.0	5.2±0.8	< 0.001
eGFR (ml/min/1.73m^2^)	68.7±7.6	87.0±15.1	< 0.001
Serum uric acid (mg/dl)	6.24±1.41	5.59±1.33	< 0.001
Time-mean serum uric acid (mg/dl)	6.39±1.29	5.64±1.28	< 0.001
Hyperuricemia (%)	81 (35.7)	836 (16.9)	< 0.001
Hypertension (%)	103 (45.4)	746 (15.1)	< 0.001
Diabetes (%)	25 (11.0)	173 (3.5)	< 0.001

*P value by T-test for continuous variables and chi-square test for categorical variables. *BMI* body mass index, *eGFR* estimated glomerular filtration rate, *BUN* blood urea nitrogen, *HDL* high-density lipoprotein, *LDL* low-density lipoprotein, *eGFR* estimated glomerular filtration rate, *CKD* chronic kidney disease. Data were expressed as mean ± SD, median (interquartile range), or percentage (%)

### Incidence of CKD

**Fig. 3:**
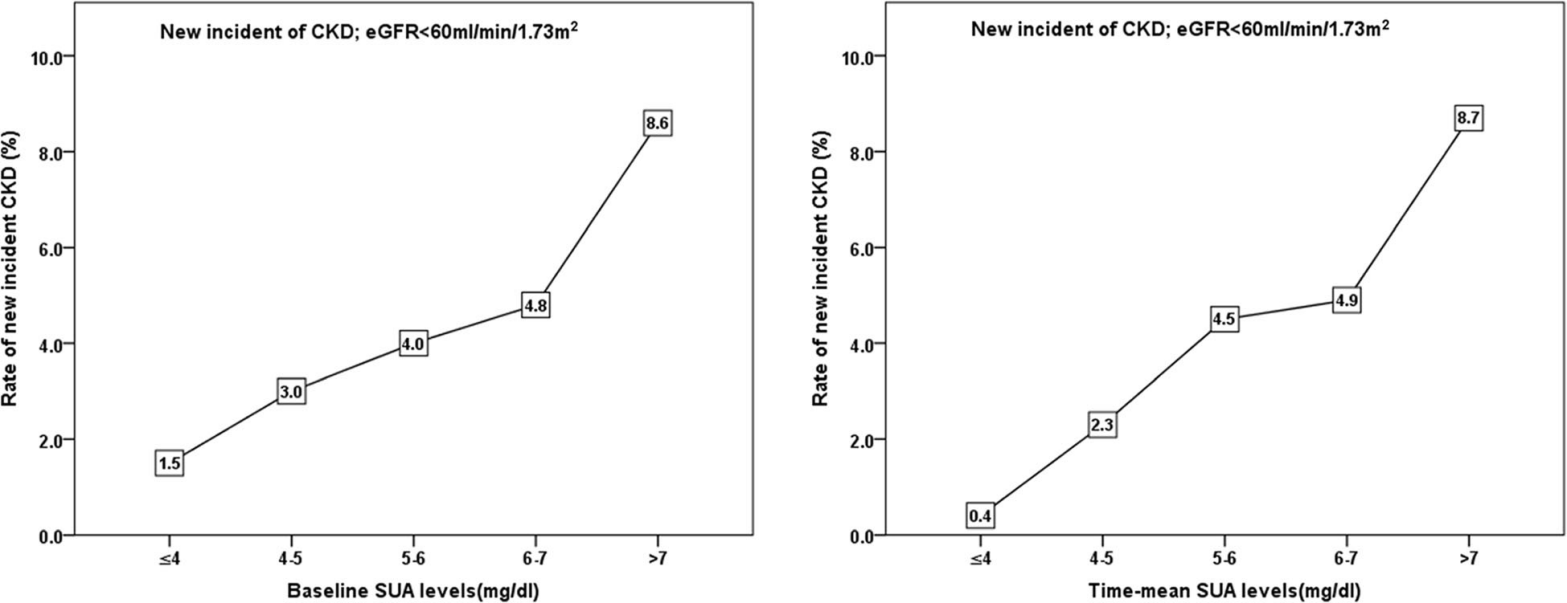
The new incidence rate of CKD for baseline uric acid and time-mean value of uric acid during follow-up period. The percentage increases significantly as baseline and mean uric acid levels increases (*p* < 0.001 by the Chi-square analysis)

Fig. 3 shows the incidence of newly developed CKD in the 6-year follow-up period. The incidence of CKD increased significantly, along with an increase in the time-mean UA levels (≤4: 0.4%, 4–5: 2.3%, 5–6: 4.5%, 6–7: 4.9%, and > 7: 8.7%, *P* < 0.001). The baseline UA levels were similarly associated with a higher incidence of CKD (≤4: 1.5%, 4–5: 3.0%, 5–6: 4.0%, 6–7: 4.8%, and > 7: 8.6%, *P* < 0.001).

### Incidence of CKD and SUA levels, using stepwise adjustment

**Fig. 4:**
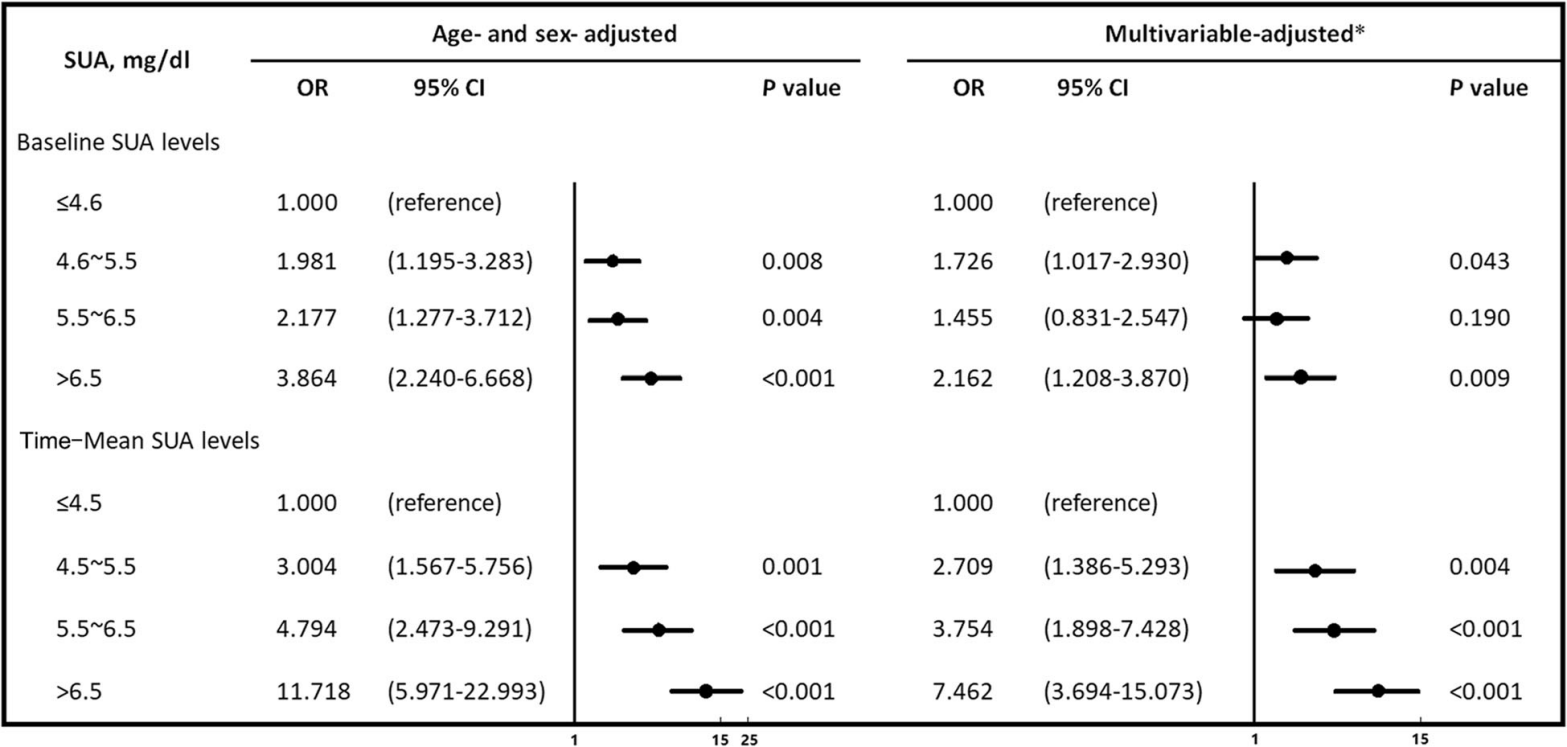
Age- and sex- adjusted and multivariable-adjusted OR for the development of CKD according to SUA levels. * Adjusted for age, sex, BMI, SBP, DBP, Total cholesterol, Baseline eGFR, FPG, Hyperuricemia, Hypertension, Diabetes. Odds ratio (OR) with their 95% confidence intervals (95% CI) for eGFR < 60 ml/min/1.73 m^2^. The first serum uric acid quartile was considered as reference category

In the age- and sex-adjusted analyses, the likelihood of developing CKD increased along with the increase of the UA levels (the odds ratio [OR] and 95% confidence interval [CI] for the Q2, Q3, and Q4 groups were 1.981 [1.195–3.283], 2.177 [1.277–3.712], and 3.864 [2.240–6.668], respectively, *P* < 0.05), compared with Q1 as a reference (Fig. 4). However, after we adjusted for various covariates in model 2, the OR decreased to 1.726 (95% CI: 1.017–2.930) in Q2 and 2.162 (95% CI: 1.208–3.870) in the Q4 group, while it widely decreased to 1.455 (95% CI: 0.831–2.547) in Q3. In contrast, the age- and sex-adjusted OR for CKD increased more remarkably, with a rise in the time-mean SUA quartile (the OR and 95% CI for Q2, Q3, and Q4 were 3.004 [1.567–5.756], 4.794 [2.473–9.291], and 11.718 [5.971–22.993], respectively, *P* < 0.001). This association remained significant, even after further adjustments for potential confounding factors in model 2, although the OR in Q2, Q3, and Q4 groups decreased to 2.709 (95% CI: 1.386–5.293), 3.754 (95% CI: 1.898–7.428), and 7.462 (95% CI: 3.694–15.073), respectively.

Furthermore, we analyzed the association between the SUA level and CKD in a subgroup analysis that was stratified by age and sex. We used the time-mean value of SUA separately in subgroups of patients over the age of 50 and under 50. In men, the association was stronger in subjects who were older than 50 than in subjects younger than 50 (OR = 1.981, 95% CI: 1.651–2.378 and OR: 1.921, 95% CI: 1.305–2.828 respectively), and there was a greater difference in women (OR: 1.867, 95% CI: 1.462–2.385 and OR: 1.454, 95% CI: 0.466–4.532 for women older and younger than 50, respectively). The same consequence was found after an additional adjustment (OR: 1.618, 95% CI: 1.3341–1.963 and OR: 1.470: 95% CI: 0.933–2.288 in older and younger men, respectively; and OR: 1.848, 95% CI: 1.404–2.433 and OR: 0.915, 95% CI: 0.222–3.762 in older and younger women, respectively).

### SUA levels and risk of CKD

**Fig. 5:**
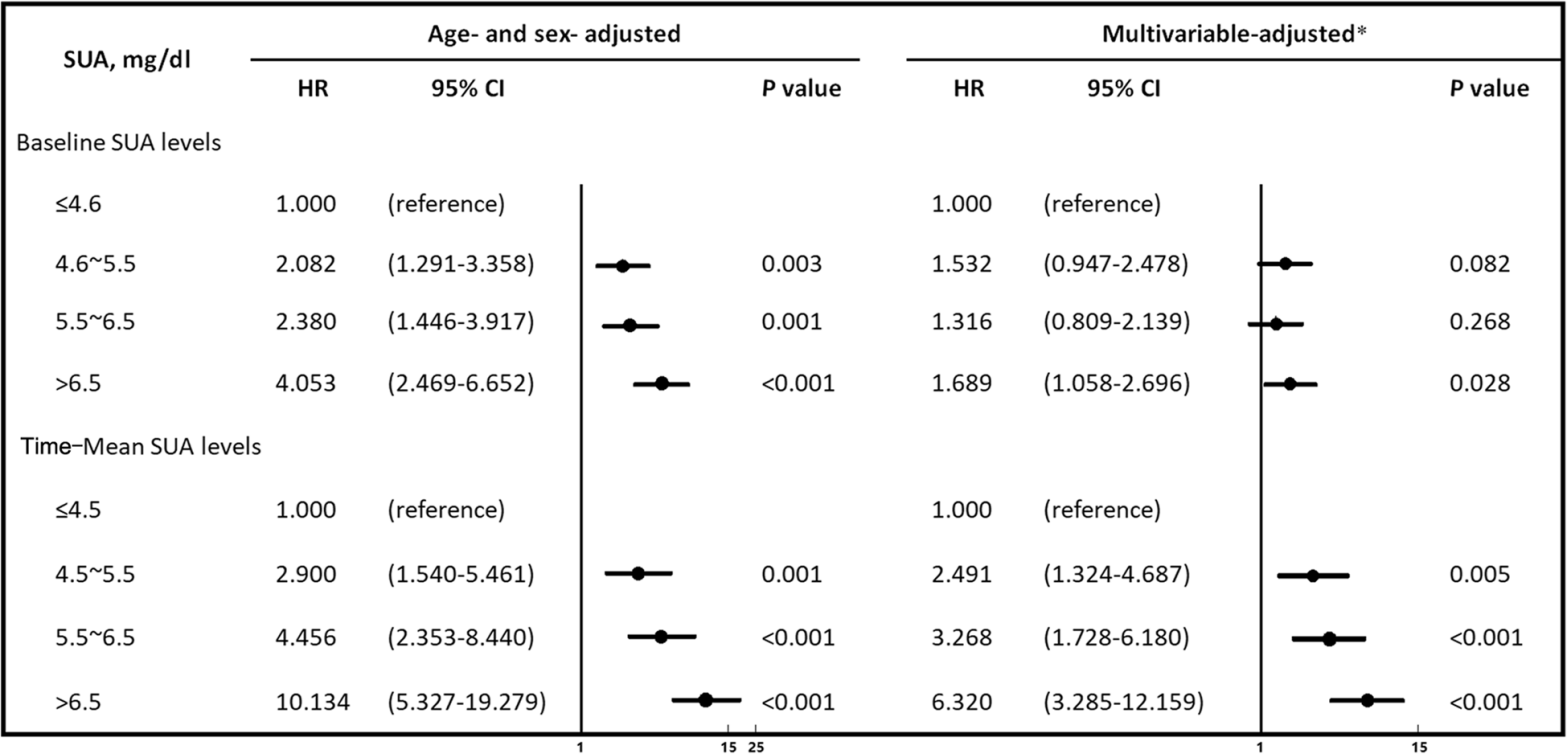
Impact of SUA levels on the risk for the development of CKD over the 6-year follow-up period. * Adjusted for age, six, BMI, SBP, DBP, Total cholesterol, Baseline eGFR, FPG, Hyperuricemia, Hypertension, Diabetes. Values were expressed as hazard ratio (95% confidence interval; HR [95% CI]) for eGFR < 60 ml/min/1.73 m^2^. The first serum uric acid quartile was considered as reference category

During the follow-up period, 227 participants developed CKD, including 139 men and 88 women (4.4 and 4.4%, respectively). As shown in Fig. 5, in the age- and sex-adjusted model, the highest baseline SUA quartile showed significant associations with the risk of CKD compared with the lowest quartile (hazard ratio [HR]: 4.053; 95% CI: 2.469–6.652, *P* < 0.05). Likewise, the highest time-mean SUA quartiles were associated with an elevated risk of CKD (HR: 10.134; 95% CI: 5.327–19.279, *P* < 0.001). After further adjusting for potential confounders, however, we found that the direction of the association was no longer the same. The strength of the association was attenuated in the baseline SUA level, and the HR for Q2 was 1.532 (95% CI: 0.947–2.478), 1.316 for Q3 (95% CI: 0.809–2.139), and 1.689 for Q4 (95% CI: 1.058–2.696). By contrast, the time-mean value of UA was still statistically significant. The HR for Q2 was 2.491 (95% CI: 1.324–4.687), 3.268 for Q3 (95% CI: 1.728–6.180), and 6.320 for Q4 (95% CI: 3.285–12.159).

## Discussion

We demonstrated that high levels of SUA are associated with the occurrence of CKD, while low SUA levels are not. Importantly, the major results of our study demonstrate that high SUA levels, particularly the time-mean SUA values, indicate the risk of renal progression and renal dysfunction after adjustment for confounding variables, and this association was even observed in the normal range of SUA levels.

It is controversial whether SUA plays a causal role in the progression of CKD or if it is merely a marker of renal damage. Many observational studies indicated that hyperuricemia is an independent risk factor for the development and progression of renal disease in healthy individuals [19–22]. However, other prospective observational studies have produced contrary results; they did not show that the SUA level had a positive effect on the incidence of CKD in Japanese patients [24–26]. On the other hand, Sturm et al. revealed that hyperuricemia predicts the progression of CKD, but only before correction. After adjusting for baseline kidney function parameters such as baseline eGFR and proteinuria, they found that hyperuricemia no longer acted as a risk factor for the progression of CKD [25].

Our findings suggest that the risk of renal function reduction might increase with increased SUA levels, especially the time-mean SUA level, even those within the normal range. First, in contrast with other studies on patients with CKD in other countries, in Chinese patients, the influences of high SUA levels on the natural history of renal function have been less examined. Our results show that in subjects who undergo annual health check-ups, the increase in the SUA level was associated with a slow decline in the eGFR, but there was a high incidence of renal insufficiency. The relationship between the SUA levels and eGFR that was observed in our study was similar to the relationship reported by a multi-center study in Japan, which included 141,514 subjects without renal insufficiency at baseline [29]. It was not similar to the relationship observed in a study by Kanda et al. in Japan [23] suggesting that the UA level has a U-shaped relationship with the loss of renal function in men, indicating that both low and high UA levels were associated with a decline in eGFR. We believe that the difference between our results and theirs [23] might be because their data were examined every 3 years, and the mean baseline UA levels in their cohort study were lower than in ours.

Second, our results revealed that high time-mean SUA levels affected the likelihood of new-onset CKD more than low levels after adjustment for confounders, while the association with baseline SUA levels was relatively milder. This is consistent with previous studies showing that elevated serum UA levels were independently associated with an increased risk of the development of CKD [20, 22, 26]. We further examined the association between the time-mean SUA values and CKD after separately evaluating participants aged > 50 years and ≤ 50 years. In both sex models, the association was present in male and female subjects > 50 years old, but absent in women ≤50 years.

Third, our analyses showed that the statistical difference was subdued in the association between the baseline SUA and incidence of CKD, but they were still statistically significant in the time-mean UA value. Participants in the highest quartile of the time-mean SUA value (> 6.5 mg/dl) had a risk of developing CKD that was more than three times higher than in those in the lowest quartile. Consistently, two studies clarified that increased SUA accelerates progression to ESRD, and indicated that the target SUA level should be less than 6.5 mg/dl [30, 31]. Similarly, Rudolf et al. found that the risk of new-onset CKD increased roughly linearly with the UA level to a level of approximately 6 to 7 mg/dl in women and 7 to 8 mg/dl in men. Above these levels, the associated risk increased rapidly [22]. Our results are consistent with those of a recent meta-analysis that included 15 cohort studies and demonstrated that there was a positive association between the SUA levels and risk of CKD in middle-aged patients, independent of established metabolic risk factors [32].

Many studies have clarified the mechanisms by which hyperuricemia leads to loss of kidney function. Animal experiments found that hyperuricemia may induce endothelial cell dysfunction and inhibit the generation of nitric oxide [12, 18]. In addition, studies have demonstrated that hyperuricemia induced arteriolopathy of the preglomerular vessels, which impairs the autoregulatory response of the afferent arterioles. Simultaneously, vascular wall thickening such as platelet adhesiveness and disturbed hemorheology [33] can result in ischemia that is induced by lumen obliteration [17]. Renal hypoperfusion is a potent stimulus of vasoactive and inflammatory mediators, ultimately leading to tubulointerstitial inflammation and fibrosis [17]. Other animal experiments demonstrated that activation of the RAAS and COX-2 systems could be mediated by upregulating the angiotensin-1 receptors on the vascular smooth muscle cells [12, 17].

The major strengths of this study were that the participants’ serum creatinine and serum UA levels were not only measured at baseline, but were also obtained every year during the subsequent follow-up period. Therefore, we could evaluate the accurate date of the onset of CKD and explore the independent effect of the time-mean SUA levels on the renal outcome. Moreover, we could avoid instability due to short-term fluctuation. Second, we analyzed the serum UA (mean ± SD: 5.6 mg/dl ± 1.3) in this cohort, which is considered representative of the Chinese general population. Third, we used the recently developed Modification of Diet in Renal Disease equation, which is known to be more accurate for Chinese people than other methods, and we adopted an eGFR of < 60 ml/min/1.73 m^2^ as the criterion to determine CKD.

Nevertheless, our study has some limitations that should be considered. First, the major limitation of our study is that the subject selection process was not entirely random. The participants were relatively healthy individuals who actively paid attention to their health status; therefore, a self-selected bias cannot be excluded in this cohort. Second, we were unable to collect data on medication history, such as UA-lowering medicines, diuretics, and antihypertensive drugs. In addition, we did not have information on lifestyle-related factors such as dietary habits, smoking, alcohol consumption, and exercise. Therefore, this study did not eliminate all the factors that might impact changes in the SUA levels and inhibit the progression of CKD.

## Conclusions

In conclusion, we observed that increased time-mean and single SUA levels are associated with a decline in renal function or even an increased likelihood of the development of new-onset CKD in the general population, after adjusting for confounding factors. Importantly, a slight increase in the time-mean SUA levels within the normal range also had a more significant predictive value in the development of kidney dysfunction than a single measured value. These findings help to emphasize the importance of regularly monitoring and managing the SUA level to delay decline in kidney function. However, studies that focus on the value of time-mean SUA to predict CVD are required.

## Abbreviations

BMI: Body mass index
BUN: Blood urea nitrogen
CI: Confidence interval
CKD: Chronic kidney disease
COX-2: Cyclooxygenase-2
CVD: Cardiovascular disease
DBP: Diastolic blood pressures
eGFR: Estimated glomerular filtration rates
ESRD: End-stage renal disease
FPG: Fasting plasma glucose
HDLC: High-density lipoprotein cholesterol levels
HR: Hazard ratio
IQR: Interquartile range
KDIGO: Kidney Disease: Improving Global Outcomes
LDLC: Low-density lipoprotein cholesterol levels
MDRD: Modification of Diet in Renal Disease
OR: Odds ratio
RAAS: Renin-angiotensin-aldosterone system
SBP: Systolic blood pressure
Scr: Serum creatinine
SD: Standard deviation
SUA: Serum uric acid
